# Multiplatform Metabolomics: Enhancing the Severity Risk Prognosis of SARS-CoV-2 Infection

**DOI:** 10.1021/acsomega.4c02557

**Published:** 2024-11-06

**Authors:** Vinicius S. Lima, Sinara T. B. Morais, Vinicius G. Ferreira, Mariana B. Almeida, Manuel Pedro Barros Silva, Thais de A. Lopes, Juliana M. de Oliveira, Joyce R. S. Raimundo, Danielle Z. S. Furtado, Fernando L. A. Fonseca, Regina V. Oliveira, Daniel R. Cardoso, Emanuel Carrilho, Nilson A. Assunção

**Affiliations:** † Programa de Pós-Graduação em Medicina Translacional, Departamento de Medicina, Escola Paulista de Medicina, Universidade Federal de São Paulo, São Paulo 04023-062, Brazil; ‡ Instituto de Química de São Carlos, Universidade de São Paulo, São Carlos 13566-590, Brazil; § Instituto Nacional de Ciência e Tecnologia de Bioanalítica, INCTBio, Campinas 13083-861, Brazil; ∥ Departamento de Química, Universidade Federal de São Carlos, São Carlos, São Paulo 13565-905, Brazil; ⊥ Faculdade de Medicina do ABC, Santo André, São Paulo 09060-870, Brazil; # Departamento de Química, Universidade Federal de São Paulo, São Paulo 05508-070, Brazil

## Abstract

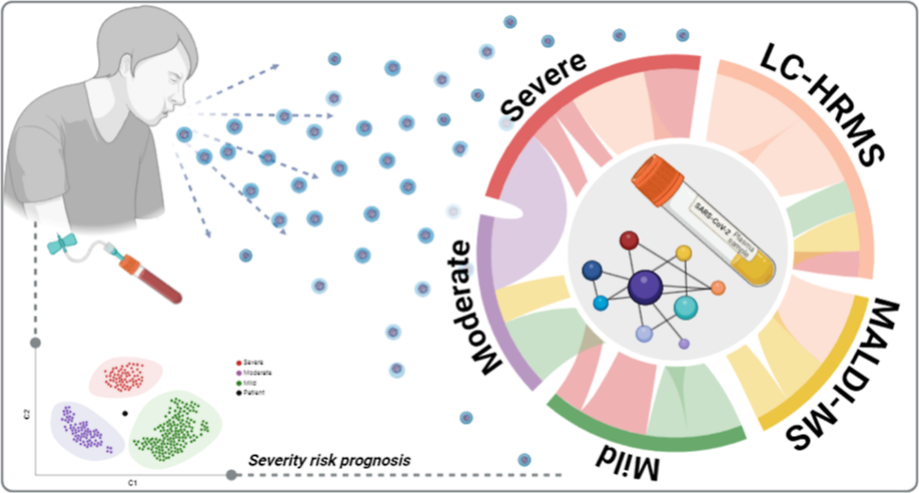

Concerns about the SARS-CoV-2 outbreak (COVID-19) continue to persist even years later, with the emergence of new variants and the risk of disease severity. Common clinical symptoms, like cough, fever, and respiratory symptoms, characterize the noncritical patients, classifying them from mild to moderate. In a more severe and complex scenario, the virus infection can affect vital organs, resulting, for instance, in pneumonia and impaired kidney and heart function. However, it is well-known that subclinical symptoms at a metabolic level can be observed previously but require a proper diagnosis because viral replication on the host leaves a track with a different profile depending on the severity of the illness. Metabolomic profiles of mild, moderate, and severe COVID-19 patients were obtained by multiple platforms (LC-HRMS and MALDI-MS), increasing the chance to elucidate a prognosis for severity risk. A strong link was discovered between phenylalanine metabolism and increased COVID-19 severity symptoms, a pathway linked to cardiac and neurological consequences. Glycerophospholipids and sphingolipid metabolisms were also dysregulated linearly with the increasing symptom severity, which can be related to virus proliferation, immune system avoidance, and apoptosis escaping. Our data, endorsed by other literature, strengthens the notion that these pathways might play a vital role in a patient’s prognosis.

## Introduction

Coronavirus disease 2019 (COVID-19), caused by SARS-CoV-2 (severe acute respiratory syndrome 2), continues to impact global health. The clinical signs of the infection can vary greatly and have already resulted in millions of deaths, emphasizing the importance of tracking the pandemic trajectory, estimating infection incidents, adequate public health planning, and, most importantly, constantly improving knowledge.^1,2^

Cough, fever, disorientation, and respiratory symptoms have been the most common in noncritical cases, ranging from mild to moderate.^3^ In severe cases, however, the infection can affect vitals, resulting in pneumonia, impaired kidney and heart function, and inpatient mortality, necessitating care and hospitalization.^4^ Consequently, understanding the interaction between the virus and the host, locating the main infection pathways, and defining potential biomarkers for diagnosing and prognosis of severe cases are critical information for disease management.^5−7^

High-resolution mass spectrometry has provided unique and specific knowledge of metabolic pathways in various disorders, characterizing a status signature and providing new potential targets for differential diagnosis. Small molecule masses can be precisely measured using MS techniques, utilizing a small sample size and providing accurate information for COVID-19 diagnosis and classification of diagnosed cases.^8^ Therefore, the type of ionization and mass analyzer are essential processes in MS since they determine the higher or lower detection sensibility of lipids and metabolites, for example.^9−11^

Metabolic deviations during a disease typically become apparent before the clinical signs. Through metabolomics, compounds with molecular weights smaller than 1 kDa can be detected and quantified, allowing the exploration of their relationship to pathological changes.^12^ Lipids and amino acids are still the main classes related to pathways impacted by the virus. Many glycerophospholipids are frequently observed in plasma cohort studies, indicating that the energetic metabolism via the tricarboxylic acid cycle is well characterized.^3,13−17^ Despite the COVID-19 era’s fast advances, metabolomics and lipidomics are still relatively young and challenging fields of study. The scientific literature lacks many biomarkers for the early identification and progression of the illness. Thus, plasma samples were analyzed using omics platforms to identify potential diagnostic and prognostic biomarkers as well as gain more insight into the metabolic effects of COVID-19, assisting with understanding the immune responses coactivated during the infection to elucidate the subclinical differences between patients diagnosed with COVID-19 who had mild, moderate, or severe symptoms.

## Material and Methods

### Patients and Sample Collection

For metabolite analysis, plasma samples from 50 people diagnosed with COVID-19 and aged 22 to 86 were included. They were collected at the ABC Foundation in Santo André, São Paulo State, a social health organization. These patients were divided into three categories, separated by disease severity: mild (18), moderate (20), and severe (10). All study participants were tested for COVID-19 infection following the WHO guidelines, using reverse transcriptase polymerase chain reaction and/or immunological blood assays to attest to IgG or IgM antibodies^18^

### Ethical Approval

The Federal University of São Paulo (UNIFESP) research ethics committee approved and received this study after it was created in compliance with the Declaration of Helsinki on the ethical conduct of research involving human participants (Bibbins-Domingo et al., 1999).^200^

### Consent to Participate

All participants received information about the study and agreed to the collection of their samples.

### LC-HRMS Sample Preparation

For the LC-HRMS-based metabolomics, 300 μL of methanol was added to 75 μL of plasma, which promoted protein precipitation and viral inactivation. Next, the precipitated samples were centrifuged at 10,000 rpm for 10 min at 4 °C, followed by the separation of 200 μL of the supernatants from each sample, of which 10 μL were isolated to make up for the quality control (QC), resulting in a total volume of 190 μL per sample. Control blanks were prepared by replacing the plasma sample with 75 μL of Milli-Q water. Finally, the materials were stored at −80 °C for later analysis.

### MALDI-MS Sample Preparation

The plasma samples were submitted to an adapted Folch method for MALDI-MS-based metabolomics. Initially, a mixture of 225 μL of methanol, 450 μL of chloroform, and 187.5 μL of water was added to 30 μL of plasma. Next, the samples were shaken for 10 min, and 300 μL of the lipidic phase was separated. For the MALDI plate preparation, a 5 μL aliquot of the lipidic extract was dried under vacuum (SpeedvacR Concentrator model SPD131DDA-115, Thermo Fischer) and fast-frozen at −80 °C until analysis. Each aliquot was solubilized in 1.5 μL of a cold solution containing 30:30:40 (v/v/v) isopropanol, acetonitrile, and methanol, followed by homogenization by pipetting at room temperature. To compensate for the signal variability among samples, PG-D5 (1-hexadecanoyl-2-(9Z-octadecanoyl)-*sn*-glycero-3-phospho-(1′-rac-glycerol-1′,1′,2′,3′,3′-d5) ammonium salt, Avant Polar Lipids Inc.) was spiked into the samples at a final concentration of 5 μM and used as an internal standard for data normalization. QC samples were prepared by pooling 5 μL of the samples and measured at the start of the batch and then every ten samples to check the experimental stability during the MS data acquisition. The matrix was made from a solution of 4-chloro-a-hydroxycinnamic acid (10 mg mL^–1^) and 2,5-dihydroxybenzoic acid (10 mg mL^–1^) in 70:30 (v/v) methanol and deionized water with a 0.1% final concentration of formic acid. Each lipid sample was mixed with the matrix solution at a 1:1 (v/v) ratio, spotted onto a MALDI-ground steel target plate with 384 sample positions (Bruker Daltonics, Bremen, Germany), and air-dried at room temperature.

### Data Acquisition

Two different techniques were used to analyze the extracted samples. The hydromethanolic extract was analyzed using an Agilent 1290 Infinity II UHPLC (Agilent Technologies, Santa Clara, CA, USA) composed of a binary pump (G7120A), autoinjector, column compartment (G7129B—1290 Vial sampler) coupled to a high-resolution time-of-flight (QTOF) mass spectrometer (Impact HD QTOF, Bruker Daltonics, Bremen, Germany), with a positive-mode electrospray ionization source (ESI+). The chromatographic separation was performed on an Agilent Zorbax Eclipse XDB-C18 reversed-phase column (100 × 3.0 mm; 3.5 μm) kept at 40 °C. The injection volume was 5 μL, and the autosampler temperature was maintained at 15 °C. The total run time was 30 min at 400 μL/min using the following multistep gradient: 0 min, 1% B; 0–3.0 min, 1–2% B; 3–10 min, 2–30% B; 10–15 min, 30–50% B; 15–18 min, 50–80% B; 18–20 min, 80–90% B; 20–22 min, 90–95% B; 22–26 min, 95–99% B; 26.01–28 min, 99% B, for column cleaning, and 28.01–30 min, 1% B, for column re-equilibration. The RPLC mobile phase consisted of 0.1% formic acid in water (solvent A) and 0.1% in acetonitrile (solvent B).

The lipidic extract was analyzed by MALDI (TOF/TOF) (AutoFlex Max, Bruker Daltonics, Bremen, Germany) with a 10 kHz smart-beam pulsed Nd/YAG laser (third harmonic, 355 nm), operating in positive ion reflector mode over the mass range of 100–1500 Da. Spectra were generated by shooting the laser at random positions and summing 2000 single spectra with the laser frequency of 2 kHz.

### Data Analysis and Preprocessing

LC-HRMS data were acquired in centroid mode using the QTOF Control Software v3.4, and the raw data were processed using the Data Analysis software and the Bruker Compass Profile Analysis 2.1 software (Bruker Daltonics GmbH, Bremen, Germany). The following settings were used for bucket generation considering the definition of molecular peaks and utilizing alignment by time: S/N threshold = 2, correlation coefficient threshold = 0.2, minimum compound length = 10 spectra, and smoothing width = 1. All features were detected with a retention time ranging from 0 to 25 min and mass-charge ratio (*m*/*z*) ranging from *m*/*z* 49 to 1301. The inclusion of features was based on absolute values of ≥5% of the absolute values obtained from the blank samples, the relative standard deviation (RSD %) of QC samples of ≤25%, and missing values of ≥30% in experimental samples. The data were normalized using the Lowess normalization software v1.1, which normalizes the variances of MS signal intensities as a function of QC analyses. Missing values were estimated using the k-nearest neighbors’ algorithm (KNN—sample-wise) in MetaboAnalyst v5.0. For LC-HRMS data analysis separately, the data was pareto scaled before statistical analysis.

MALDI spectra were processed by the Flex Analysis software (Bruker Daltonics, Bremen, Germany), where external calibration was conducted by a peptide standard mixture (Peptide Calibration Standard II, Bruker). Further procedures were carried out by a set of library packages, MALDIquant and MALDIrppa, on R software to improve signal quality and compress the raw data into a list of relevant *m*/z values. All the raw data were smoothed using a moving average filter algorithm, and the baseline was corrected using the statistics-sensitive nonlinear iterative peak-clipping algorithm (SNIP). All peaks over a signal-to-noise ratio threshold of 3 using the Super Smoother algorithm were identified as relevant *m*/*z*. Peaks were aligned and binned with a tolerance of 0.05 Da, and only peaks present in more than 40% of all samples were retained for further analysis, avoiding artifacts and noise contributions. To generate the final intensity data set, features with *m*/*z* values below 300 were excluded due to interference from background peaks. For MALDI data analysis separately, data was additionally normalized by mean, log transformed, and pareto scaled before statistical analysis.

### Multiplatform Data Integration

**Figure 1:**
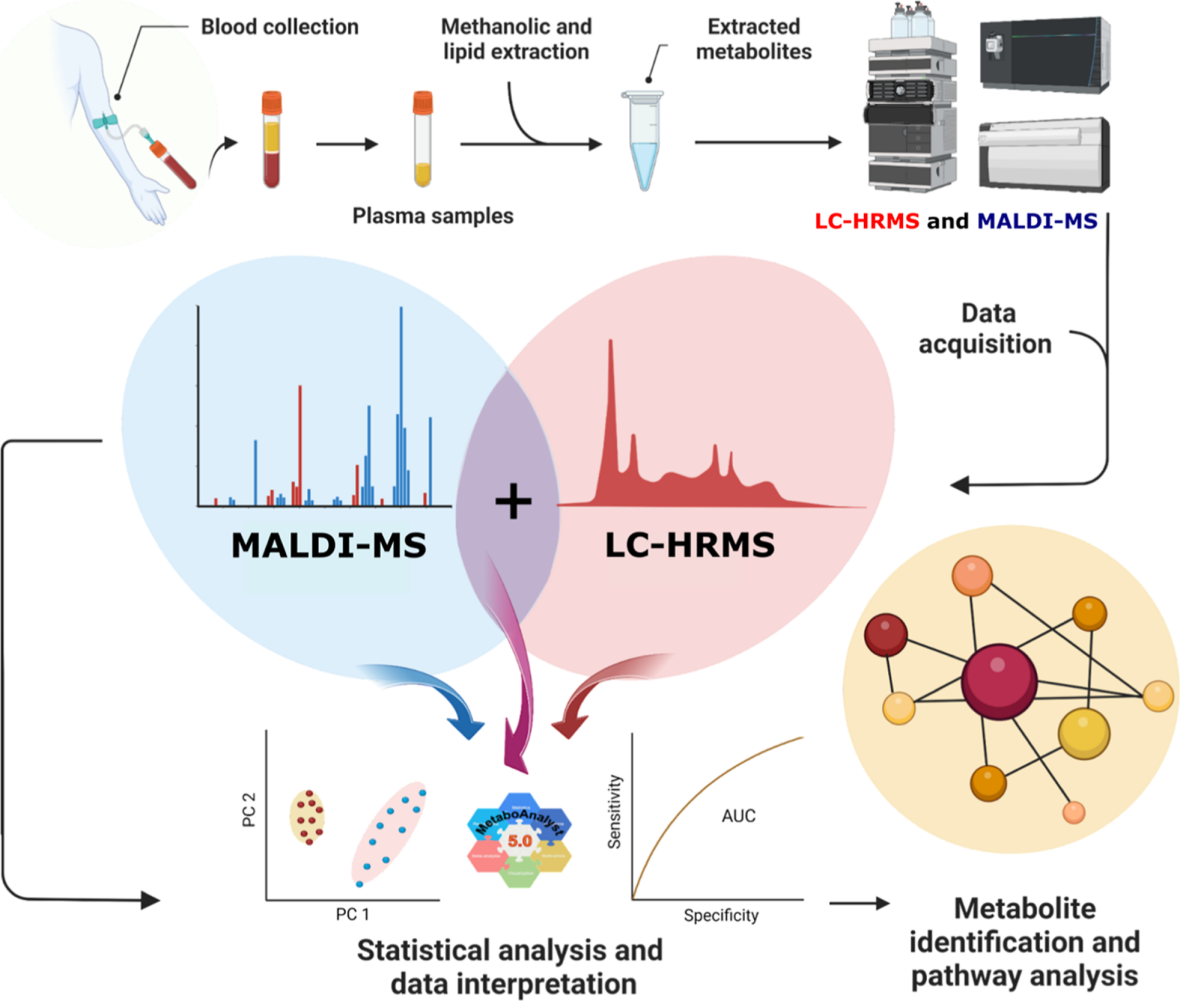
Multiplatform analysis workflow for data acquisition and two sample extraction protocols performed for lipidic and methanolic extracts, respectively. The extracts were analyzed using two different analytical approaches: LC-HRMS for the methanolic extract and MALDI-MS for the lipidic extract. Initially, the data were analyzed separately and combined to build a single statistical model. Finally, the most critical metabolites were annotated, identified, and submitted to a metabolic pathway analysis. Created in BioRender. Carrilho, E. (2023) BioRender.com/e20g315.

Before integrating the LC-HRMS and MALDI data, each platform’s data was evaluated independently, which include comprehensive data preprocessing, univariate, and multivariate analyses. The preprocessing steps, suited to each platform, were carried out as described above. After confirming the data quality independently for each platform, the LC-HRMS and MALDI significant features were combined into a single data set. The merge was performed on preprocessed data, using the initial normalizations specific to each platform (Lowess normalization for LC-HRMS to address batch effects, and internal standard normalization for MALDI to account for instrumental variation). Only significant features from different analyses were combined, 17 from LC-HRMS and 39 from MALDI analysis, respectively. Once integrated, the resulting matrix was autoscaled to ensure that metabolites from both platforms were handled equally in subsequent studies (Figure 1).

### Statistical Analysis

Principal component analysis (PCA), ANOVA (Kruskal–Wallis test), and partial least-squares discriminant analysis (PLS-DA) were performed on the Metaboanalyst v5.0 platform. Leave-one-out cross-validation (LOOCV) and variable importance for the projection (VIP) scores were utilized to evaluate the predictive capacity and identify key features of the model. Receiver operating characteristic (ROC) curves and permutation tests were also conducted to assess the potential of the features as biomarkers. Various performance indicators, including accuracy, sensitivity, and specificity were evaluated, with 95% confidence intervals.

### Features Annotation and Identification

Metabolites annotation was carried out by searching fragmentation pattern data deposited on public databases such as the Human Metabolome Database (www.hmdb.ca), Metlin (https://metlin.scripps.edu/), MassBank (https://massbank.eu/), and LIPIDMAPS (www.lipidmaps.org). To reveal any matching, MS/MS fragmentation spectra of the statistically significant features were compared to the most likely hits based on accurate MS.

Annotated metabolites were evaluated against pre-established human metabolome pathways deposited in the Kyoto Encyclopedia Gene and Genome (https://www.kegg.jp/), allowing the identification of the most altered pathways and their functional impact.

## Results

Researchers are still furthering their understanding of the virus’s effects on human metabolism. Through the past years, many papers have been published regarding the metabolic effects of the SARS-CoV-2 virus, accessing such information by diverse analytical platforms.^19−30^ In our work, we decided to apply the reliability of conventional LC-HRMS for the analysis of high and medium-polarity metabolites, combined with the high throughput of MALDI-MS for nonpolar metabolite analysis, to evaluate the plasma samples from positive cases of COVID-19 at different clinical conditions. Each platform generated a high-dimensional data set, providing complementary information.

**Figure 2:**
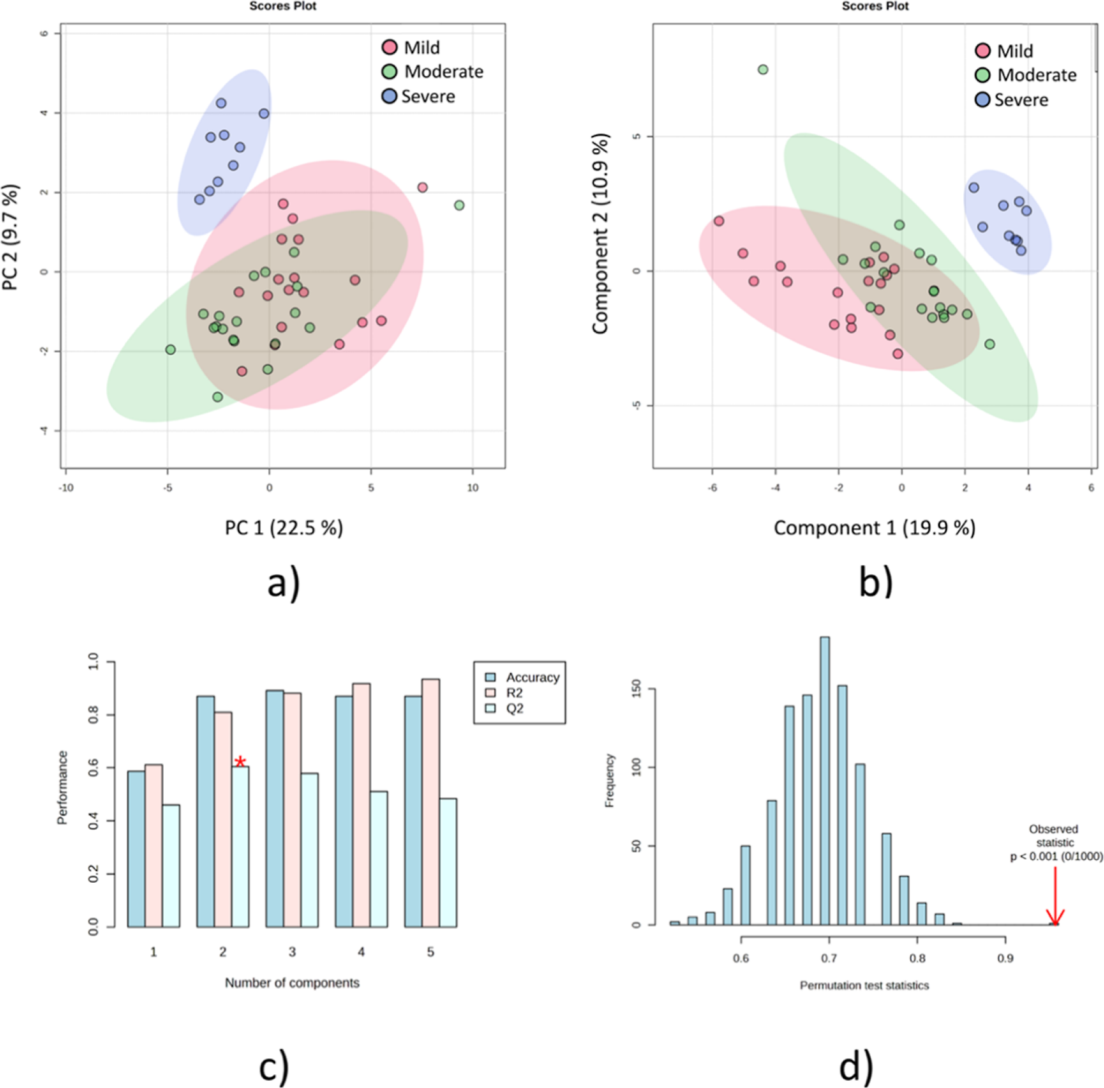
Multivariate statistical analysis from multiplatform analysis of mild, moderate, and severe symptom groups (a) PCA analysis of the three analyzed groups; (b) PLS-DA analysis; (c) cross-validation model of the PLS-DA analysis; and (d) permutation test with 1000 permutations of the PLS-DA analysis.

To ensure data reliability and quality, specific preprocessing and separate data analysis were performed for each platform. The preprocessing steps and analysis workflows were carefully carried out based on established protocols from relevant literature.^31−34^ PCA and Partial-Least Squares Discriminant Analysis (PLS-DA) of each platform separately can be seen on Figures S1 and S6, respectively. PCA analysis contains QC sample information, and their clustering demonstrates a low level of analytical variability. PLS-DA analysis (Figure S1) showed a clear tendency of separation between the symptom’s severity levels (mild, moderate, and severe) for each platform separately. The analysis pointed to relevant features (*m*/*z*) obtained using both platforms. PCA, as an unsupervised approach, can be considered unbiased, and the group separation formed by the score’s plots can be related to the loadings, which indicate features responsible for the group’s separation, and this information was used as a first variable selector. Adding class label information on the samples through a PLS-DA provided information about discrepancies between and within groups (Figure S1). The data were carefully checked for overfitting, and the quality assessment (Q^2^) was higher than 0.4 for both platforms, a value considered acceptable for biological models.^31^ Subsequent analysis combined statistically significant features identified from LC-HRMS (19) and MALDI-MS (40) for a full data interpretation as a multiplatform data set (Figure 2).^32,35^

Following the steps outlined in Figure 1, new PCA plots for the multiplatform data set were generated after data autoscaling (Figure 2a). Besides the fact that the PCA separation has not been able to separate the groups in two dimensions, the 3D plot (Figure S2a) showed a clear separation tendency, highlighting the relevance of the selected features in the analysis by the individual platforms. Notwithstanding, PLS-DA was performed to construct a model to separate the severity groups and emphasize the most critical features for group discrimination (Figure 2b). Cross-validation (Figure 2c) and the permutation test (Figure 2d) of the PLS-DA model resulted in a Q^2^ of 0.60 and a p-value <1 × 10^–4^, evidencing the model’s quality and statistical relevance.

**Figure 3:**
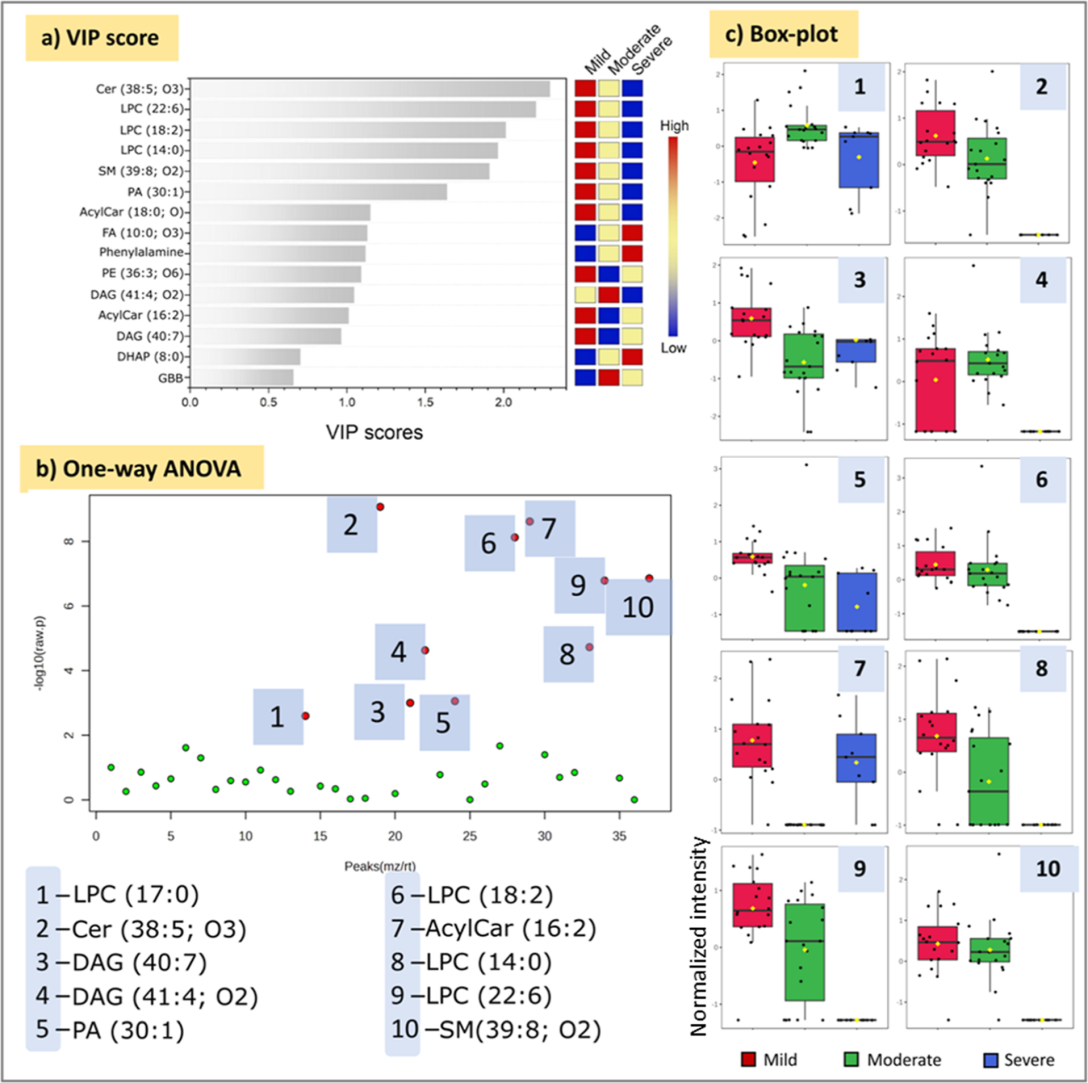
Most important features for severity group’s discrimination obtained by multiplatform approach. (a) Important features ranked based on PLS-DA VIP score analysis; (b) important features based on One-Way ANOVA test; and (c) box-plot for selected features (normalized values).

From the PLS-DA analysis, a VIP graph was obtained, overviewing and ranking six metabolites as the most important features (VIP > 1.5) for group discrimination (Figure 3a). Surprisingly, the levels of such metabolites decrease as the severity of the symptoms increases. A one-way ANOVA analysis was performed to access the relevant metabolites by univariate analysis (Figure 3b). Regardless of the statistical method, the highlighted metabolites (*p* < 0.05) were the same.

**Figure 4:**
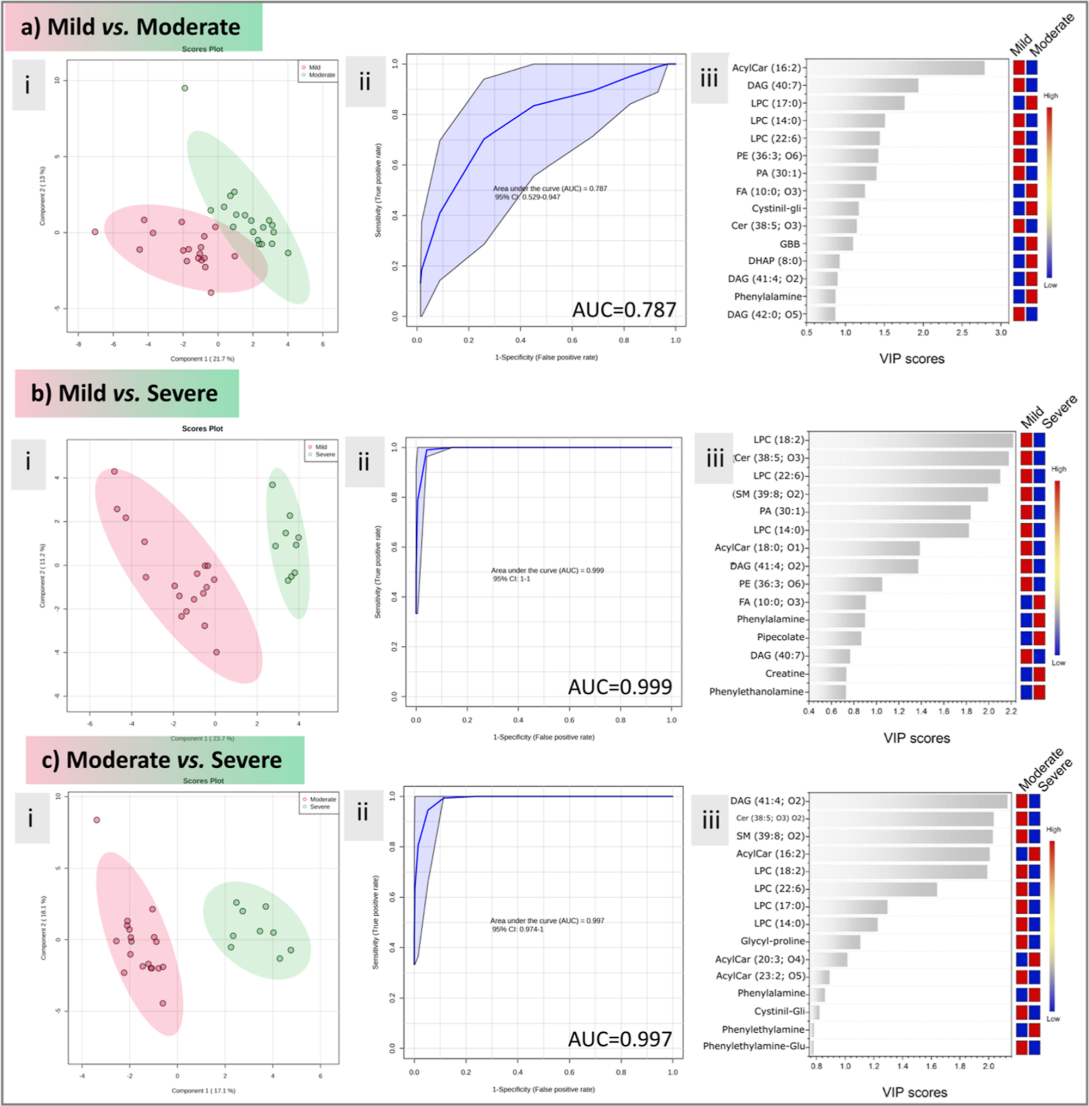
Binary comparisons between mild vs moderate (a), Mild vs Severe groups (b) moderate vs severe (c) groups showing PLS-DA (i), ROC curve (ii), and VIP scores (iii) plots.

Binary comparisons were performed to assess better the specific impact of COVID-19 in the discrimination of the severity groups (Figure 4). The results suggest a similar behavior between mild and moderate symptom metabolism since, besides a good separation in the PLS-DA analysis and a good prediction coefficient (Figure S3), the generated ROC curve presented a low AUC (0.787). Notwithstanding, the PLS-DA group separation and ROC curve prediction (Figure 3) were highly favorable for the mild vs severe and moderate vs severe comparisons (Q^2^ > 0.7 and AUC > 0.99 for both cases) (Figures S4 and S5). As expected, the same metabolites were important for group differentiation; however, with this analysis, we could correctly validate such metabolites’ ability to classify patients from mild to severe symptoms. Such information can be beneficial in predicting prognosis in patients with early COVID-19 symptoms.

**Figure 5:**
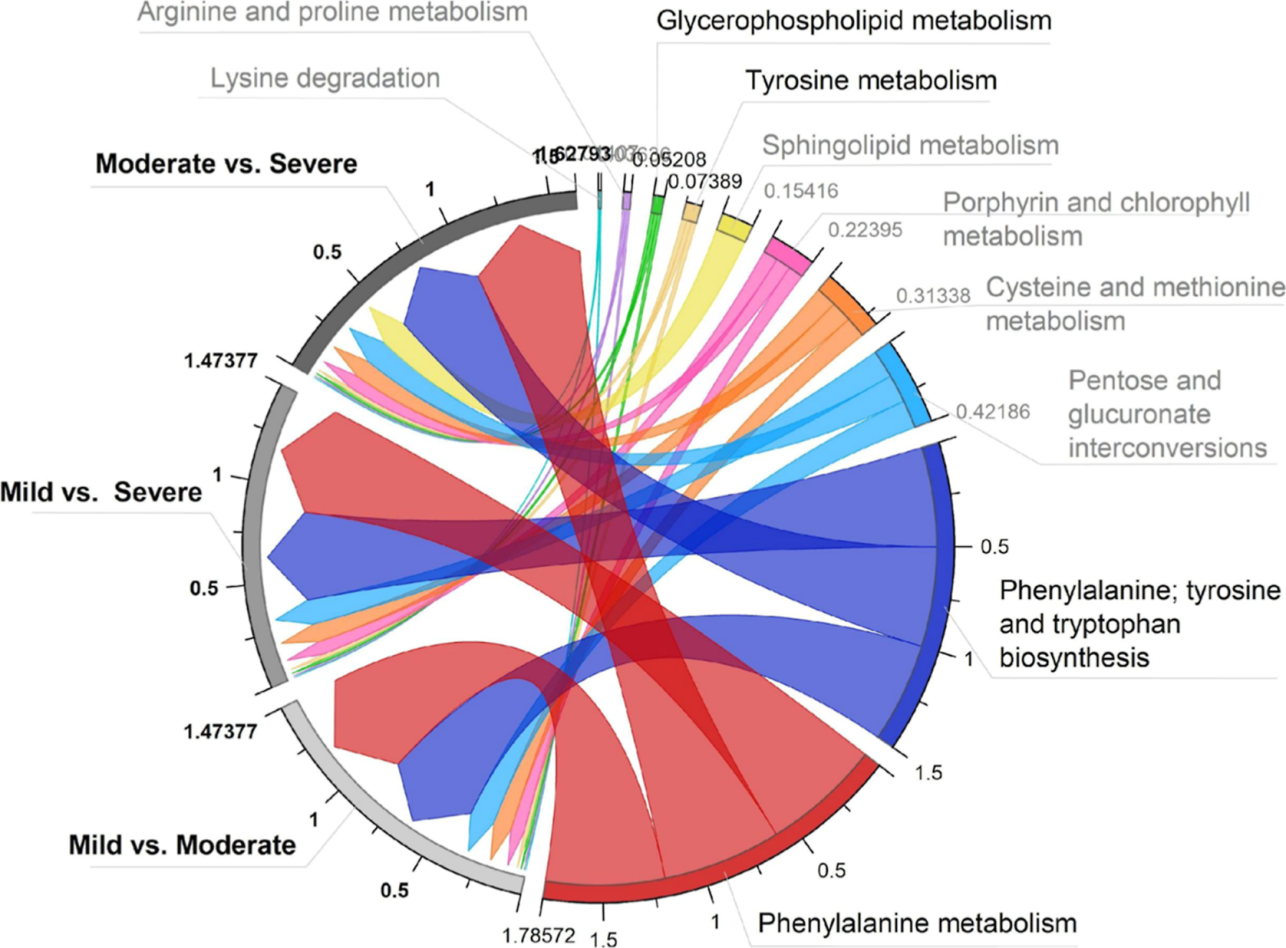
Chord plot of the impacted metabolic pathways. The comparison between symptom intensities is presented on the left side of the chord plot, while the affected metabolic pathways are shown on the right side. Pathways with *p* < 0.05 are highlighted.

A chord plot was generated using the impacted values obtained during the analysis to understand better the association between the metabolic pathways acting in the change of severity status (Figure 5). It is easily recognized by the size of the chord originating from the right side (metabolic pathway) and going straight to the impacted groups (left side). The scale represents the impact value (summary) of the metabolic pathways. The data set provides ten pathways impaired by the SARS-CoV-2 infection. Significant pathways (*p* < 0.05) with ascending impact values included glycerophospholipid metabolism, tyrosine metabolism, phenylalanine, tyrosine, and tryptophan biosynthesis, and phenylalanine metabolism. Except for SM metabolism, which was only observed in moderate vs severe cases, most of the pathways had some association with all of the binary comparisons.

## Discussion

On its own, omics data contributes significantly to the scientific knowledge related to variations associated with a specific illness. But, in medicine and biology, integrating multiplatform data sets with clinical information has been much more groundbreaking. Biomolecules from many origins, all available from a single source, can reveal the biochemical history of the system under investigation and fill in the gaps around the circumstances created by certain illnesses.^36,37^

Considering this scenario, this study uses two independent platforms to build a prediction model for COVID-19 symptom evolution based on expanded metabolomic coverage. The polar and medium-polarity metabolites were analyzed using LC-HRMS, whereas the nonpolar metabolites were accessed promptly using MALDI-MS. Our findings support the roles of phenylalanine and glycerophospholipid metabolism as key pathways for understanding the severity of COVID-19 symptoms, not only building on previous work from our group but also expanding on and emphasizing the importance of lipids in symptom severity classification in COVID-19 infection.^25^ Furthermore, the relevance of MALDI-MS for metabolic study is noteworthy since such a technique has an exceptional high-throughput capability for metabolic fingerprinting, especially when compared to traditional metabolomics methods such as LC-HRMS and NMR.

### Phenylalanine Role in COVID-19

The metabolic profile of individuals with severe COVID-19 symptoms indicated elevated phenylalanine levels in a previous study we published utilizing qNMR-based metabolomics.^25^ As expected, the phenylalanine (Phe), tyrosine (Tyr), and tryptophan (Trp) biosynthesis revealed alterations in the group’s binary comparison by COVID-19 severity status (Figure 5). However, in this paper, the multiplatform approach did not include quantification results. Still, it is possible to notice the importance of this pathway for mild vs severe and moderate vs severe cases. Phenylalanine is one of the essential amino acids, and researchers have been reporting its high levels in cases of infections, indicating metabolic disturbances that could lead to death.^38^ According to the impact pathway analysis in Figure 5, statistically significant values of 0.5 for both severity statuses affected by Phe, Tyr, and Trp biosynthesis and 0.6 for Phe metabolism observed for moderate vs severe cases indicate that the assumption of high Phe levels is possibly related to infection severity.

Different papers have correlated phenylalanine and infection severity.^39−43^ The presence of high levels of Phe in the plasma may indicate a malfunction of the enzymes Phenylalanine 4-hydroxylase (PHA) and 5,6,7,8-tetrahydrobiopterin (BH4), which are in charge of converting Phe to Tyr.^39^ During infections, the host-infected cells produce reactive oxygen species (ROS) as an initial response, triggering the innate immune system by releasing proinflammatory cytokines and activating the NF-kB pathway.^43,44^ However, the elevated concentration of ROS may damage the cellular enzymes, such as PHA and BH4, leading to an increase in Phe levels in the host organism. The concentration can also be enhanced by the catabolism of the destroyed host cells.^40^ Either way, phenylalanine levels are highly associated with infection severity and could be responsible for additional disease symptoms, such as cognitive outcomes, psychiatric symptoms, and aberrant behavior.^45−48^

Although psychological illnesses developed as a result of the pandemic’s stressful and unpleasant experiences, the direct consequences of the virus infection on the brain have been widely described.^49^ Current research on post-COVID-19 disorders discovered a large percentage of individuals with long-term cognitive impairment, including symptoms such as brain fog, memory issues, headache, and depression. These illnesses can still be identified after six months.^49,50^ This research aims not to link metabolic changes to post-COVID-19 symptoms; nonetheless, it is worth noting that increased Phe levels may be linked to cognitive impairment, like what is seen in phenylketonuria (PKU) patients.^51^ The elevated Phe level in PKU leads to reduced bioavailability of Try and Trp, the building blocks for serotonin and dopamine synthesis.^52^ Downregulation of neurotransmitters, in turn, affects synaptic transmissions in the central nervous system, altering the patient’s cognitive function.^53^

Like in sepsis and HIV-1 infections, elevated levels of Phe and disruption of its associated pathways in COVID-19 patients may be associated with a higher likelihood of cardiovascular events, albeit the mechanism is not entirely understood.^54,55^ As previously stated, elevated Phe levels are associated with increased ROS and immune cytokines like IL-8 and IL-10, which could cause muscle tissue damage, including cardiac tissue.^56,57^ Numerous investigators reported a strong link between heart failure mortality and high Phe levels.^56,58^ SARS-CoV-2, on the other hand, causes severe damage to numerous organs, debilitating the patient and increasing susceptibility to opportunistic infections, which can lead to a generalized infection and death. The elevated Phe levels should not thus be utilized as a predictor of mortality. Wang et al. discovered a genetic component in cardiac patients that might be activated by stress, resulting in hyperphenylalaninemia.^59^ As a result, elevated levels of Phe in cardiac patients would be the result of a genetic predisposition rather than an infection-related upregulation. In that case, high Phe in COVID-19 patients could be used as a prognostic biomarker for increased risk for cardiac complications, one of the leading symptoms in severe and long-term COVID-19.

### Bioactive Lipids Impact in COVID-19

The lipid level disturbances in COVID-19 patients attracted attention among the metabolites due to their role in cellular regulatory circuits.^60−64^ Using host lipids on virus membranes allows the virus to bypass the host immune system, allowing for smoother replication and a higher viral load.^13^ Therefore, lipids have been related to acute respiratory distress syndrome and sepsis in severely ill patients.^65,66^

In our study, COVID-19 patients showed a disturbance in the metabolism of glycerophospholipids and SMs, the last being the major class of lipids in eukaryotic cells.^64^ Glycerophospholipids (GPs) are known to be used by RNA viruses to construct their viral replication complexes, and glycerophospholipid metabolism reprogramming is closely related to viral replication.^67^ SMs, on the other hand, are known to modulate proliferation, differentiation, and apoptosis in human cells.^68^ In that sense, its downregulation may prevent infected cells from entering apoptosis, allowing the virus to keep increasing. SM metabolism had an impact value ten times higher than the GP metabolism pathway, the GP being the only route assigned only for one binary comparison, moderate vs severe cases, suggesting their importance for the virus and, as a consequence, a worsening of the clinical status (Figure 5). In-depth, among the GPs, the phosphatidylcholines were the most downregulated in the COVID-19 patients. These lipids are essential components of biomembranes, playing important roles as signal transduction mediators and immune activation processes.^69^ Downregulation of this lipid class had previously been reported for discriminating between negative and positive COVID-19 groups, and it was also linked to liver damage.^3,13,70−72^ Enveloped RNA viruses, such as SARS-CoVs, depend on the host’s lipids. In this regard, the downregulation of glycerophospholipids in COVID-19 patients remains part of the lipid rearrangement and manipulation of lipid metabolism to assist viral entry and promote virus infection and replication. Thus, to produce the lipids needed to construct specialized vesicles for viral RNA synthesis, such as fatty acids and lysophospholipids, GP is hydrolyzed by a cytoplasmatic phospholipase (PLA2), resulting in suppressed levels of these metabolites in positive samples.^73^ This enzyme has been considered a potential key factor in coronavirus replication and may be investigated as a therapeutic target.^73^

Most coronavirus symptoms are related to vascular integrity loss, which affects vital organs, including the lungs, heart, and brain.^74^ SMs, important components of cell membranes, are involved in several cell processes, including signaling pathways, that can subvert many of the severe complications of COVID-19.^75,76^ Interestingly, we found a downregulation of SMs aligned with COVID-19 progress and severity (Figure 3c). The literature has already described this feature as associated with COVID-19 patients in critical conditions.^13,77^ In this regard, recent and prior literature provides ample evidence that SM metabolism may play a role in viral infection management and vascular integrity maintenance.^78−82^ Additionally, SMs have been reported to play important roles in activating and modulating inflammatory responses by promoting macrophage activation and migration to inflammatory sites and inhibiting apoptosis.^76,83,84^ Thus, disrupting the balance in SM metabolism may impact their immunomodulatory effect and the management of the syndrome’s complications and side effects.

Our data show that acylcarnitines (AcylCar) were found at higher levels in patients who had severe symptoms, which is consistent with the literature (Figures S3d, S4d, and S5d).^22,77,85^ These compounds, essential for fatty acid delivery to mitochondria for oxidation and energy production, had already been associated with lung injury and COVID-19’s worst prognosis.^86−88^ Previous studies also reported increased levels of this metabolite in COVID-19 patients, including the deceased group, pointing out that the levels of most acylcarnitines returned to normal values after hospital discharge.^22,77^ Exacerbated accumulation of acylcarnitine was also related to inhibition of the ability of pulmonary surfactants to prevent alveolar collapse, compromising lung function, and reducing oxygen capacity in respiratory syndromes such as influenza.^13,88^ Other authors suggested that this accumulation may inhibit ion channels, disrupt calcium signaling, and impair ATP production.^89^

In conclusion, our findings indicate a significant connection between phenylalanine metabolism and increased COVID-19 severity symptoms, which can also be linked to increased cardiac and neurological implications. Glycerophospholipid and SM metabolisms were similarly dysregulated linearly as the intensity of the symptoms increased, which might be associated with viral growth, immune system avoidance, and apoptosis escape. This work demonstrates how such pathways and metabolites might be predictive biomarkers for COVID-19 symptoms. Therefore, further validation is required for an accurate biomarker evaluation.

## Supporting Information Available

Further statistical analysis is provided. Metabolomics statistical analysis for both LC-HRMS and MALDI-MS platforms, separately, are presented. Additionally, validation metrics for the PCA and PLS-DA models are provided, including cross-validation, permutation tests, and correlation heatmaps, for the comparison between mild, moderate, and severe cases (PDF)
