# Supplementary material for: Multiplatform Metabolomics: Enhancing the Severity Risk Prognosis of SARS-CoV-2 Infection
Source: ACS Omega. 2024 Nov 6;9(46):45746–58. doi: 10.1021/acsomega.4c02557 (PMC11579725; doi:10.1021/acsomega.4c02557)
Supplement: Supplementary file 1 — ao4c02557_si_001.pdf [file ao4c02557_si_001.pdf]

Multi-platform metabolomics: enhancing the severity risk prognosis of SARS-CoV-2 infection

Vinicius S. Lima,[1†] Sinara T. B. Morais,[2†] Vinicius G. Ferreira,[2,3†] Mariana B. Almeida,[2,3†], Manuel Pedro Barros Silva[1†], Thais de A. Lopes,[4] Juliana M. de Oliveira,[4] Joyce R. S. Raimundo,[5] Danielle Z. S. Furtado,[1] Fernando L. A. Fonseca,[5,6] Regina V. Oliveira,[4] Daniel R. Cardoso,[2] Emanuel Carrilho,[2,3*] Nilson A. Assunção,[1,6*]*

[1] Programa de Pós-Graduação em Medicina Translacional, Departamento de Medicina, Escola Paulista de Medicina, Universidade Federal de São Paulo, São Paulo, SP, Brazil.
[2] Instituto de Química de São Carlos, Universidade de São Paulo, São Carlos, SP, Brazil.
[3] Instituto Nacional de Ciência e Tecnologia de Bioanalítica, INCTBio, Campinas, SP, Brazil.
[4] Departamento de Química, Universidade Federal de São Carlos, São Carlos, SP, Brazil.
[5] Faculdade de Medicina do ABC, Santo André, SP, Brazil.
[6] Departamento de Química, Universidade Federal de São Paulo, São Paulo, SP, Brazil.
[†] These authors contributed equally to this work.

*Corresponding authors:
Nilson A. Assunção - nilson.assuncao@unifesp.br
Emanuel Carrilho - emanuel@iqsc.usp.br
Daniel R. Cardoso - drcardoso@usp.br

1. Supporting Information

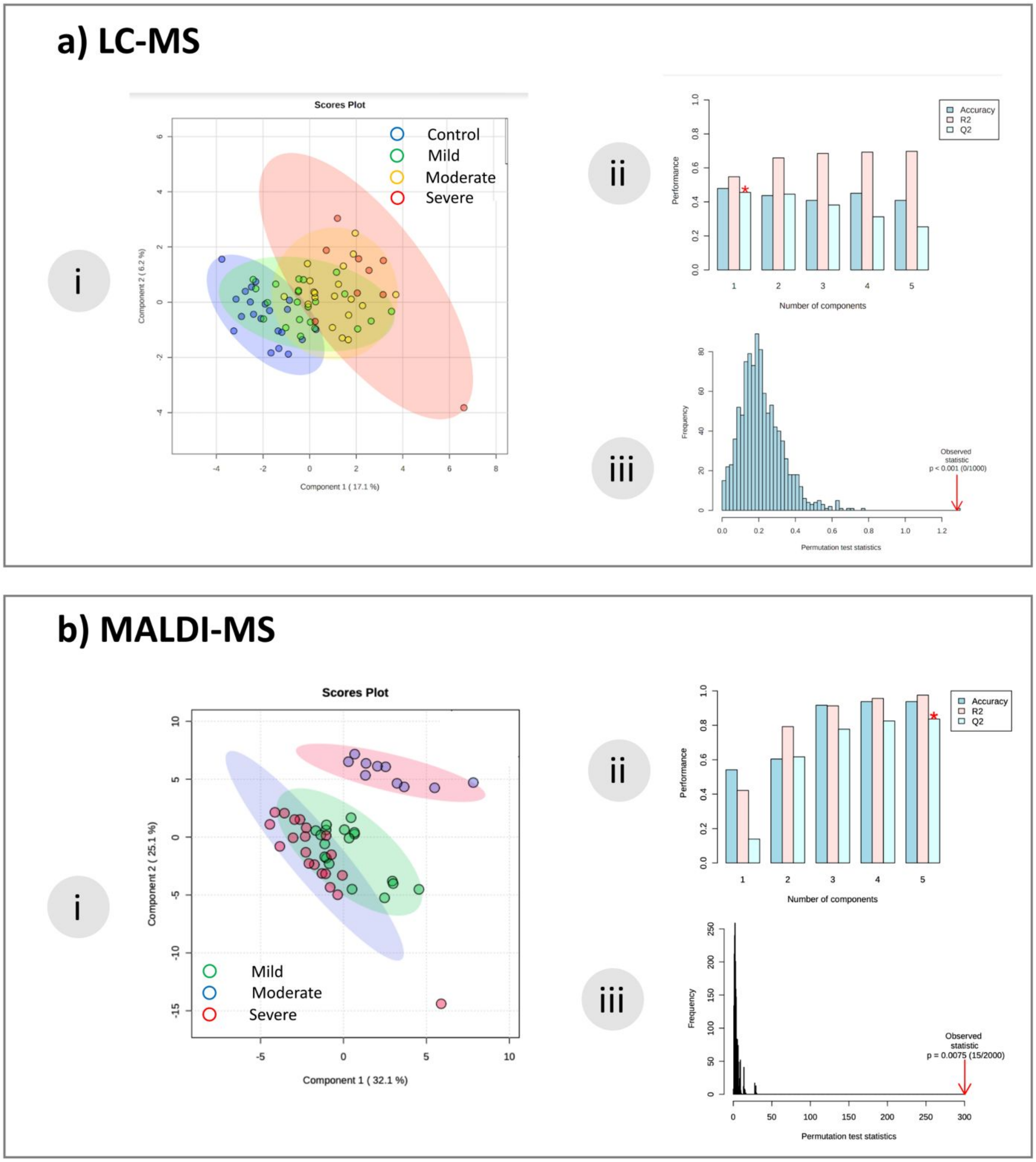

Figure S1. Statistical analysis for a) LC-MS and b) MALDI-MS datasets. The figure displays the plots of PLS-DA (i), cross-validation (ii), and permutation test (iii).

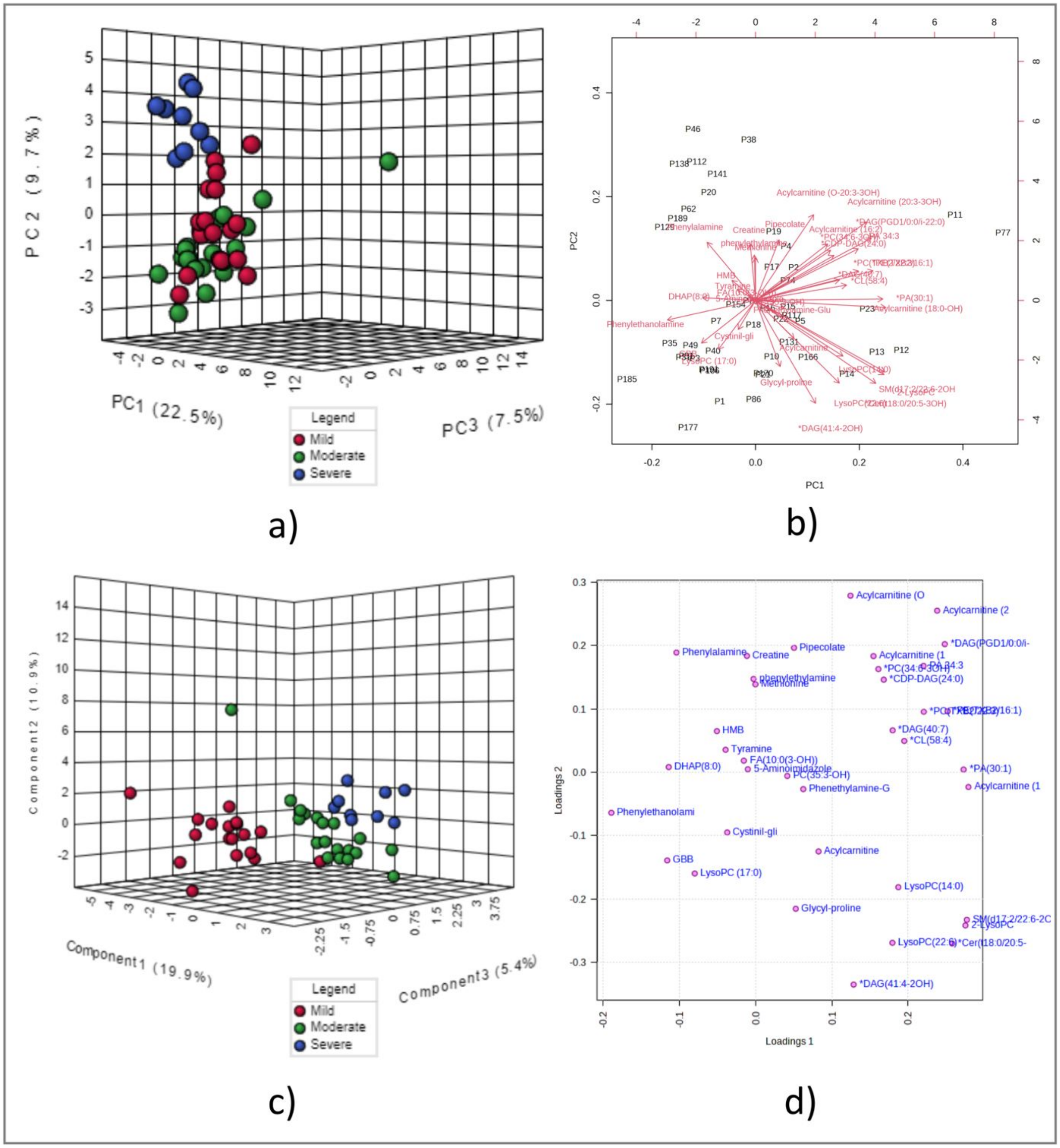

Figure S2. Multi-platform statistical analysis: 3D PCA (a) and biplot (b); 3D PLS-DA (c) and loadings plot (d).

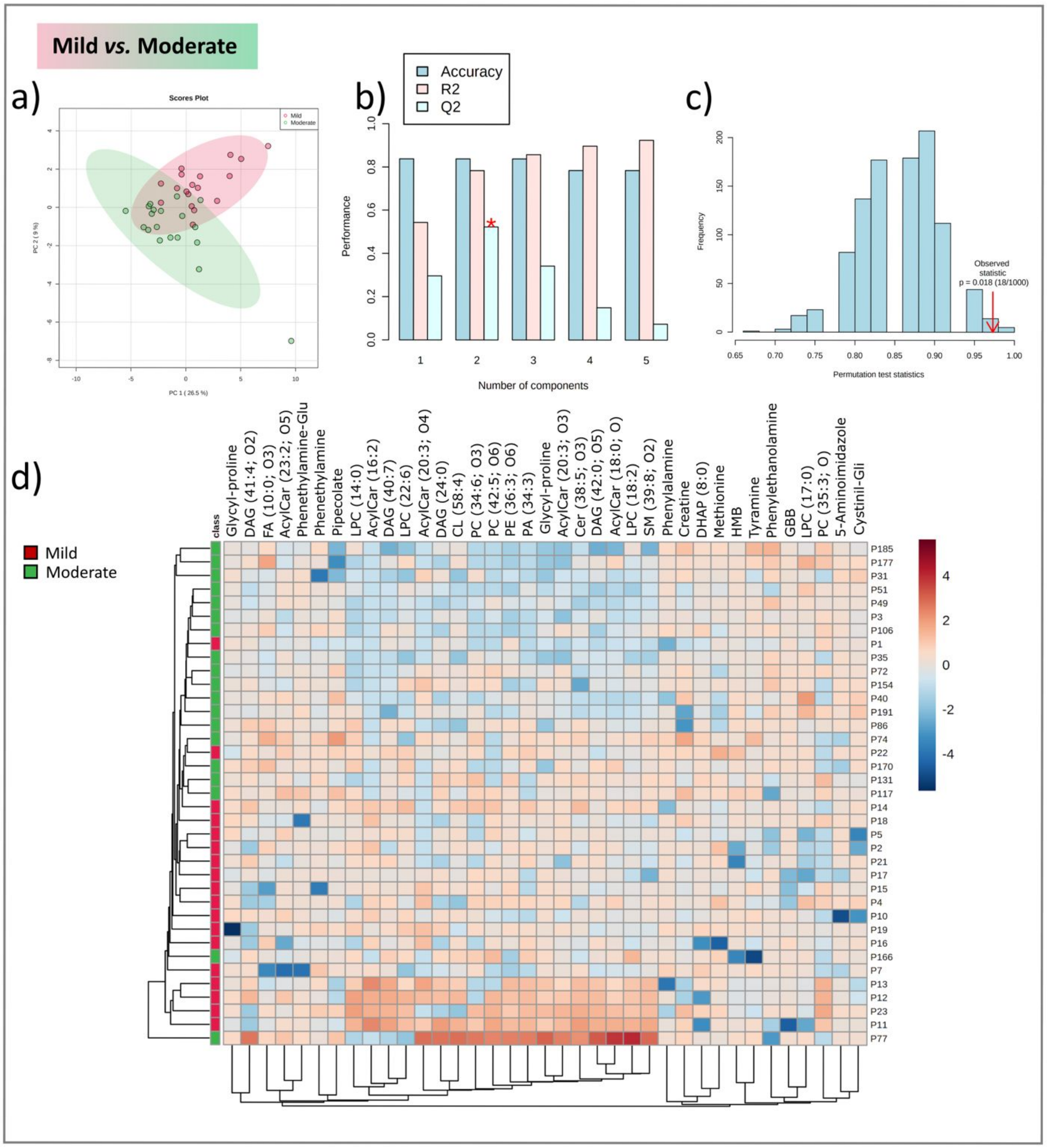

Figure S3. Mild *vs.* Moderate PCA and PLS-DA validation metrics: a) PCA, b) cross-validation, c) permutation test, and d) heatmap.

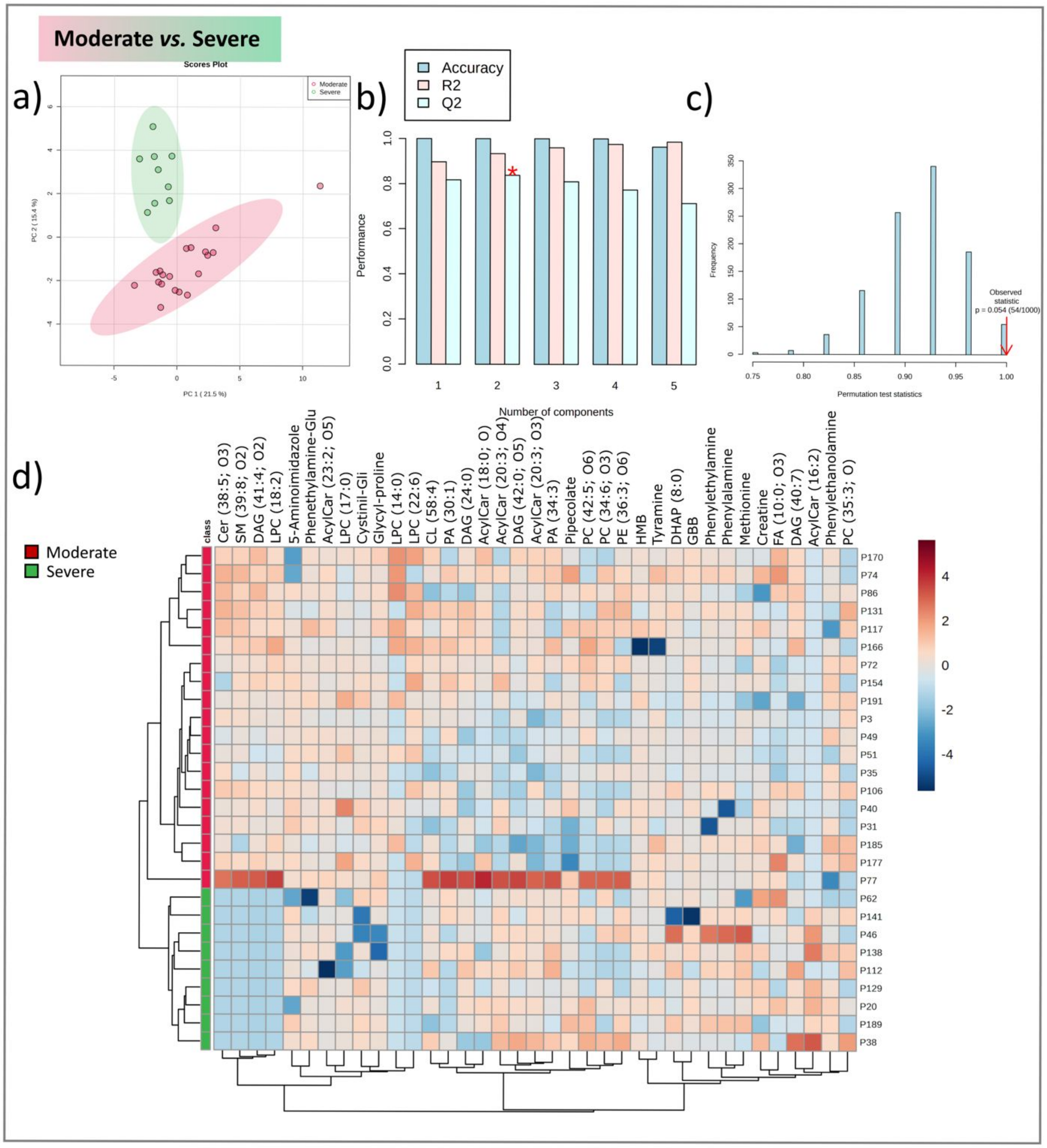

Figure S4. Moderate *vs.* Severe PCA and PLS-DA validation metrics: a) PCA, b) cross-validation, c) permutation test, and d) heatmap.

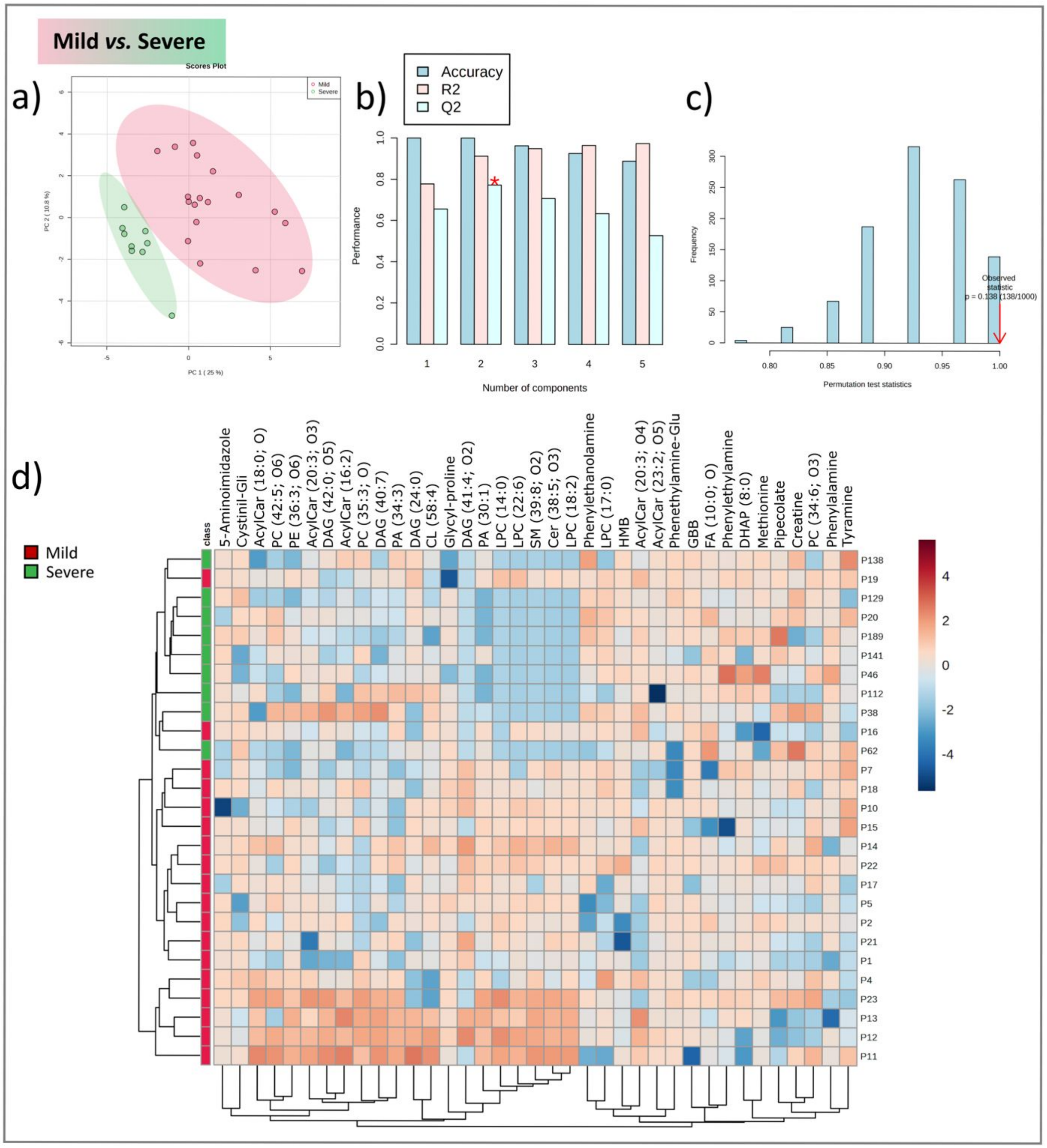

Figure S5. Mild *vs.* Severe PCA and PLS-DA validation metrics: a) PCA, b) cross-validation, c) permutation test, and d) heatmap.

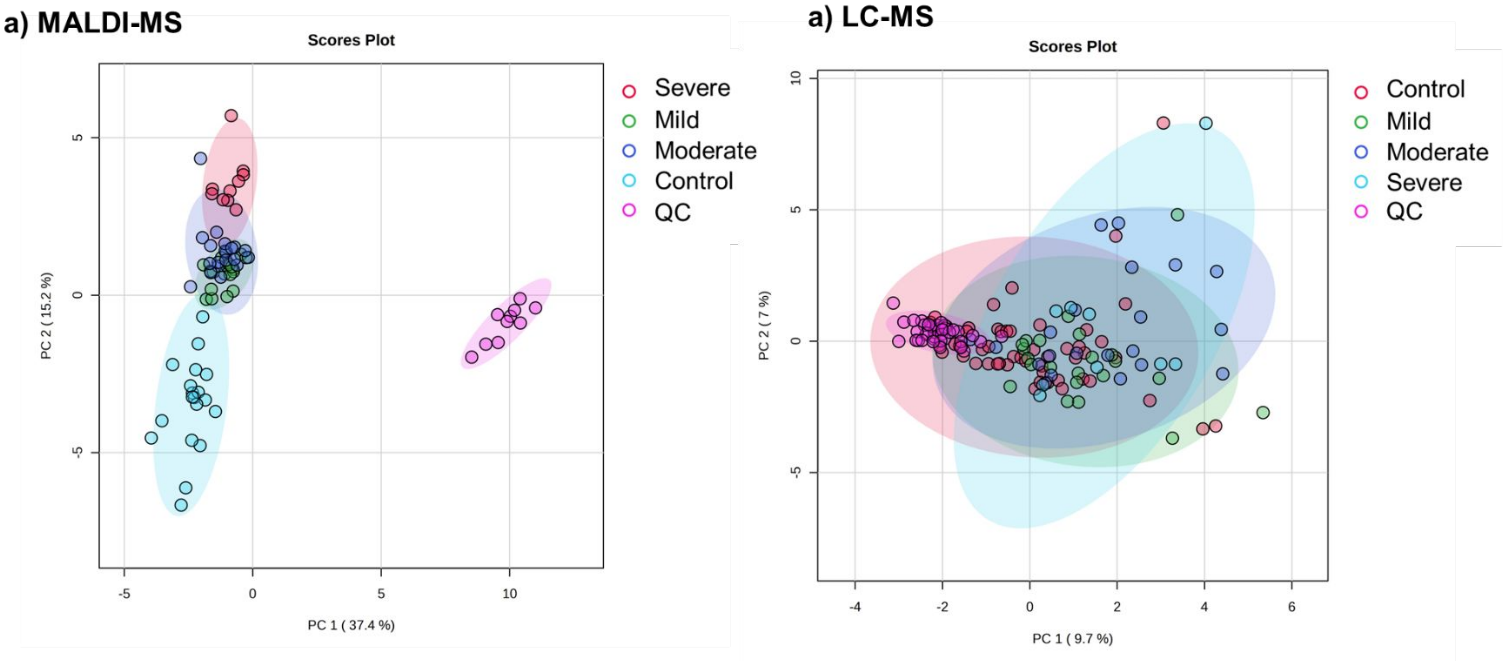

Figure S6. PCA Scores Plot analysis of LC-MS and MALDI-TOF data separately.
